# A review of the enhanced CJD surveillance feasibility study in the elderly in Scotland, UK

**DOI:** 10.1186/s12877-023-04556-z

**Published:** 2024-01-03

**Authors:** Lovney Kanguru, Sarah Cudmore, Gemma Logan, Briony Waddell, Colin Smith, Anna Molesworth, Richard Knight

**Affiliations:** 1 National CJD Research & Surveillance Unit (NCJDRSU), Centre for Clinical Brain Sciences, University of Edinburgh, Western General Hospital, Edinburgh, EH4 2XU UK; 2 NHS Lothian and Queen Margaret University, Musselburgh, Scotland; 3 Department of Neurology, Ninewells Hospital, Dundee, Scotland; 4 Edinburgh Brain Bank (EBB), Centre for Clinical Brain Sciences, University of Edinburgh, Chancellor’s Building, 49 Little France Crescent, Edinburgh, UK

**Keywords:** Creutzfeldt-Jakob Disease, vCJD, sCJD, Prion Disease, Surveillance, Public health, Health protection, Scotland, Elderly, Neurology

## Abstract

**Background:**

Variant Creutzfeldt - Jakob disease (vCJD) arose from dietary contamination with bovine-spongiform-encephalopathy (BSE). Because of concerns that vCJD-cases might be missed in the elderly, a feasibility study of enhanced CJD surveillance on the elderly was begun in 2016. Recruitment was lower than predicted. We describe a review of the challenges encountered in that study: identification, referral, and recruitment, and the effects of actions based on the results of that review.

**Methods:**

Review was conducted in 2017. Study data for all eligible cases identified and referred from one participating service (Anne Rowling clinic (ARC)) was curated and anonymised in a bespoke database. A questionnaire was sent out to all the clinicians in medicine of the elderly, psychiatry of old age and neurology (including ARC) specialties in NHS Lothian, exploring possible reasons for low recruitment.

**Results:**

Sixty-eight cases were referred from the ARC (March 2016-September 2017): 25% were recruited. Most cases had been referred because of diagnostic uncertainty. No difference was seen between those recruited and the non-recruited, apart from age and referrer. Twelve of 60 participating clinicians completed the questionnaire: only 4 had identified eligible cases. High workload, time constraints, forgetting to refer, unfamiliarity with the eligibility criteria, and the rarity of eligible cases, were some of the reasons given. Suggestions as to how to improve referral of eligible cases included: regular email reminders, feedback to referrers, improving awareness of the study, visible presence of the study team, and integration of the study with other research oriented services. These results were used to increase recruitment but without success.

**Conclusion:**

Recruitment was lower than predicted. Actions taken following a review at 21 months did not lead to significant improvement; recruitment remained low, with many families/patients declining to take part (75%). In assessing the failure to improve recruitment, two factors need to be considered. Firstly, the initial referral rate was expected to be higher because of existing patients already known to the clinical services, with later referrals being only newly presenting patients. Secondly, the unplanned absence of a dedicated study nurse. Searching digital records/anonymised derivatives to identify eligible patients could be explored.

## Introduction

Variant Creutzfeldt - Jakob disease (vCJD) is a very rare neurodegenerative disease, one of the prion diseases, which are associated with the presence of an abnormal form of a normal protein (the prion protein), in certain body tissues. Nearly all vCJD cases have resulted from dietary contamination with bovine-spongiform encephalopathy (BSE) although secondary, human-human, transmission has occurred through blood and blood product treatments [1, 2]. To date, 178 definite and probable cases of vCJD have been reported in the United Kingdom (UK) in a single epidemic wave, with the last known case reported in 2016 with symptom onset in 2014 [3, 4]. All but one tested definite and probable cases have been methionine homozygous at codon-129 of the prion protein gene [3], however, all three codon-129 genotypes are thought to be susceptible to infection and have been identified amongst those with asymptomatic vCJD infection [1, 2, 5]. While the recognised cases are individual tragedies, their number is small compared with the likely high exposure of the UK population to BSE in diet [6].

Individuals in the pre-clinical phase of vCJD are capable of transmitting the disease, as evidenced by the known blood transfusion cases, and a permanently asymptomatic infection state may exist. From a public health perspective, the prevalence of asymptomatic BSE/vCJD infection in the UK general population is a vital consideration. It is estimated at 1 in 2000 population on the basis of studies performed on routine surgical tonsil and appendix samples [7–9]. The discrepancy between the number of dietary and secondary vCJD cases, and both the dietary exposure and the presumed prevalence of asymptomatic infection in the population, is open to several possible explanations. One of these is possible case under ascertainment, despite the existence of a mature national CJD surveillance system. Arguably, missing vCJD cases would be more likely to occur in the elderly population, where referral to specialist neurological services might take place less often, other cognitive brain diseases are much more common and brain autopsy rates low [10].

Clearly, a comprehensive diagnostic study of all dementia in the elderly, with complete autopsy data, is the only definitive way of finding any possibly missed cases of vCJD. However, this would involve unmanageable numbers and many challenges (including autopsy consent and capacity). The study reviewed here focussed on a particular patient category (‘atypical’ patients-as defined in the study methodology) in one UK health region (NHS Lothian). The study had two main aims: (1) To test the feasibility of this study design and (2) To detect any missed vCJD cases, within the limits set by the methodology. Although this second aim related particularly to vCJD, possible missed cases of all prion diseases were included, for two main reasons. Firstly, the most important differential diagnoses of vCJD is other forms of prion disease, especially sporadic CJD (sCJD), the commonest human form. Secondly, it has long been noted that the annual mortality rate for sCJD rises steeply with increasing age, but then falls off in the very elderly, and it has been proposed that this might, at least in part, reflect under-ascertainment of sCJD in that age group. This study, therefore, had the potential for answering this question. In the design of the study, calculations were made as to the expected recruitment numbers. Given that 1% of the general population are expected to develop dementia annually, of which, about 10% might be considered clinically ‘atypical’, it was anticipated that 300 cases might be recruited i.e. taking into account 80% power for detection of cases with 95% confidence intervals within a 5% margin of error.

Recruitment to the Lothian enhanced surveillance multi-site feasibility study began in 2016. The study focussed on patients aged 65 years and above, who presented with features atypical for the common dementing illnesses, or lacking a disease-characteristic profile, with consequent diagnostic uncertainty. It was noted that far fewer than expected cases were being identified, referred, and recruited and, at 21 months, a review of patient data and clinicians’ experiences was undertaken to inform actions to try to improve recruitment. The methodology and general results of the study have been described elsewhere [11]; here we discuss the review of the study and the difficulties inherent in the study’s adopted approach.

## Methods

The review was carried out in 2017 as part of the enhanced CJD surveillance feasibility study, which had received ethics approval from Scotland A Research Ethics Committee (reference ref: 15/SS/0196).

### Design

The review used two approaches. Firstly, examination of data collected (of cases referred) from one study participating site, the Anne Rowling Clinic (ARC), which provides clinical neurological care and is also a clinical research facility. When patients first attend the ARC, they are asked, as a routine, if they will give written consent to be approached for any future research studies. The ARC keeps a register of those patients who give such consent (the Edinburgh Cognitive Disorders Clinic Diagnosis, Audit, Research and Treatment Register: CDC-DART). This register was screened by the local clinical team to identify eligible cases for the 65 + enhanced CJD surveillance feasibility study. Data for all the eligible cases identified and referred to the study was curated and anonymised in a bespoke database. The ARC was selected for this approach as it contributed the greatest number of patients to the study and the data were in a bespoke database. It was considered that these data would be reasonably representative of the whole and that approaching all involved clinicians would be logistically difficult and burdensome.

Secondly, a questionnaire was developed and all the study referring clinicians from Medicine of the Elderly (MOE), Psychiatry of Old Age (POA) and Neurology (including ARC) specialties invited to participate. The questionnaire focussed on referral: identification of eligible cases, introducing the study to eligible cases, and the referral process itself, including any challenges encountered. The study nurse emailed the questionnaire to all the relevant clinicians, and, if there was no response, followed up with a reminder a few weeks later.

### Study database of cases referred from the ARC

The bespoke study database included relevant sociodemographic characteristics: sex, age at invitation, current living status, main carer and who had referred them to ARC. Also, relevant clinical characteristics such as suspected diagnosis, reason for referral (to the 65 + study), symptoms, pre-existing comorbidities, alcohol history, and number of consultations (including number of days between the last consultation visit and invitation to the study) were included. We used the most recent record for suspected diagnosis. For cases that had their last consultation visit before the actual study start date, we used the study start date as the censor date. All the information collected during the course of the review was kept in line with the NCJDRSU Data Protection and Security Code of Practice. It was kept confidential, held securely in a cloud-based healthcare trusted research environment (AIMES), and paper records locked in secure cabinets; access to personal information restricted to the study team.

### Outcomes of interest

The primary outcome of interest was, as far as possible, to determine the reasons for the lower than expected identification, referral and recruitment of cases, including any particular challenges faced by participating clinicians and patients, and to determine any specific patient characteristics that influenced the likelihood of participation or non-participation.

### Analysis

The bespoke study database was cleaned, and the data crosschecked in MS excel and MS access, and later analysed in STATA v14. Descriptive statistics were computed for independent variables of interest for both the recruited and non-recruited cases. All the characteristics are presented as numbers (%) for categorical variables, and mean (*95% Confidence Interval, Standard Deviation*) for continuous variables. We employed *X*^*2*^*tests* and *t* tests as appropriate, to explore initial links i.e. whether specific sociodemographic and clinical characteristics were linked to recruitment or not. Modelling was considered to adjust for confounding, however, due to the small numbers involved, it was not undertaken. Data from the questionnaire was analysed thematically, using NVIVO software.

### Actions

Where possible, appropriate actions were taken on the basis of the review to try to improve referral and recruitment.

## Results

In 2017, at 21 months of the study, the initial projection suggested that about 175 cases would have been identified as eligible for study inclusion; only 72 cases had been referred from the ARC and 47 from all the other services, not all meeting the eligibility criteria.

**Table 1:** Sociodemographic characteristics

	Recruited (*n* = 17)	Not recruited (*n* = 51)	P value	Overall (*n* = 68)
**Sex**			0.575	
Male	10 (58.8%)	26 (51.0%)		36 (52.9%)
Female	7 (41.1%)	25 (49.0%)		32 (47.1%)
**Age at invitation (years)**				
Mean (SD)	72.2 (5.97)	70.3 (5.20)		70.8 (5.42)
(min, max)	(65, 87)	(64, 87)		(64, 87)
**Age at invitation, stratified (years)**			0.137	
64*–69	6 (35.3)	27 (52.9)		33 (48.5)
70–74	5 (29.4)	17 (33.3)		22 (32.4)
75+	6 (35.3)	7 (13.7)		13 (19.1)
**Current living status**			0.455	
Alone	1 (5.9)	9 (17.7)		10 (14.7)
With someone	15 (88.2)	38 (74.5)		53 (77.9)
Not known	1 (5.9)	4 (7.8)		5 (7.4)
**Main carer**			0.591	
Spouse	11 (64.7)	30 (58.8)		41 (60.3)
Other family	1 (5.9)	10 (19.6)		11 (16.2)
Organised care	2 (11.8)	4 (7.8)		6 (8.8)
Not known	3 (17.7)	7 (13.7)		10 (14.7)
**Referrer**			0.677	
General Practitioner (GP)	4 (23.5)	14 (27.5)		18 (26.5)
General Adult Psychiatry	1 (5.9)	3 (5.9)		4 (5.9)
Neurology	6 (35.3)	11 (21.6)		17 (25.0)
Old Age Psychiatry	3 (17.7)	6 (11.8)		9 (13.2)
Other	2 (11.8)	6 (11.8)		8 (11.8)
Not known	1 (5.9)	11 (21.6)		12 (17.7)

SD – Standard Deviation, *there was only one case referred and invited to the study who was 64 years old but had already turned 65 years when recruited

### Study database of cases referred from ARC

The database contained data from March 2016 to September 2017. Seventy-two cases had been referred to the 65 + study during that period. For this review, four cases have been omitted from the analysis: three cases had their diagnoses confirmed through genetic testing (C9orF72 gene), and 1 case was noted to have no evidence of primary neurodegenerative disorder. Hence, sixty-eight cases were analysed in this review. Table 1 summarises the socio-demographic characteristics of all the cases identified and referred. Of these, 25% agreed to participate in the 65 + study. There was no major difference between those recruited and the non-recruited, apart from age and referred by. A higher proportion of non-recruited were in the 64 to 69 year age group (52.9%), and had been referred to ARC by their general practitioner (27.5%).

Table 2 presents clinical characteristics of all cases. In both groups, all the cases had been referred because of the following reasons: their diagnosis fell between two conditions, there was more general diagnostic uncertainty or mixed aetiology was suspected. Of those referred and recruited, the most common clinical suspicion was of Alzheimer’s Dementia (47.1%), and of those then not recruited, Frontotemporal Dementia (FTD) syndromes (33.4%). All the characteristics were not statistically different between recruited and non-recruited, except for one symptom (fluency, *p = 0.032*). However, caution should be taken in the interpretation of this result given the small numbers involved and confounding.

**Table 2:** Clinical characteristics

Suspected diagnoses at referral	Reasons for referral (all cases, N = 68)		Recruited, N = 17 (N, %)	Not recruited, N = 51 (N, %)
Alzheimer’s Disease	Diagnosis lies between two conditions (2 cases), Diagnostic uncertainty (4 cases), Mixed aetiology (1 case), Uncertain aetiology (2 cases), reason not stated (10 cases)		8 (47.1)	11 (21.6)
FTD Syndromes				
- Behavioural FTD (bvFTD)	Diagnosis lies between two conditions (3 cases), Uncertain aetiology (1 case), reason not stated (7 cases)		0	11 (21.6)
- Primary Progressive Aphasia (PPA)	Diagnosis lies between two conditions (1 case), Diagnostic uncertainty (2 cases), Uncertain aetiology (3 cases), reason not stated (1 case)		1 (5.9)	6 (11.8)
- Logopenic Aphasia	Diagnosis lies between two conditions (1 case)		1 (5.9)	0
Progressive Supranuclear Palsy	Diagnosis lies between two conditions (2 cases), Diagnostic uncertainty (3 cases), reason not stated (2 cases)		2 (11.8)	5 (9.8)
Corticobasal Degeneration	Diagnostic uncertainty (1 case), reason not stated (2 cases)		1 (5.9)	2 (3.9)
Mixed Dementia	Diagnosis lies between two conditions (3 cases), Diagnostic uncertainty (3 cases), Mixed aetiology (2 cases), reason not stated (1 case)		2 (11.8)	7 (13.7)
Posterior Cortical Atrophy	Diagnosis lies between two conditions (1 case), reason not stated (2 cases)		1 (5.9)	2 (3.9)
Lewy Body Dementia	Diagnostic uncertainty (1 case), Uncertain aetiology (1 case)		0	2 (3.9)
Vascular Dementia	Diagnostic uncertainty (1 case)		0	1 (2.0)
Unspecified Dementia	Diagnostic uncertainty (2 case), reason not stated (1 case), Uncertain aetiology (1 case)		1 (5.9)	3 (5.9)
Multiple System Atrophy	Uncertain aetiology (1 case)		0	1 (2.0)
**Other clinical characteristics**				
		**Recruited** **(*n*** **= 17) (n, %)**	**Not recruited** **(*n*** **= 51)**	**P value**
Symptoms* (pre-65 + study referral)				
- Progressive deterioration		15 (88.2)	49 (96.1)	0.202
Deficits				
◦ Memory		17 (100.0)	45 (88.2)	0.334
◦ Language		11 (64.7)	38 (74.5)	0.298
◦ Fluency		13 (76.5)	39 (76.5)	0.032
◦ Behaviour		10 (58.8)	31 (60.8)	0.672
◦ Attention		10 (58.8)	28 (54.9)	0.232
◦ Visuospatial		14 (82.4)	31 (60.8)	0.221
Pre-existing comorbidities*				
- Diabetes		1 (5.9)	6 (11.8)	0.787
- Hypertension		5 (29.4)	16 (31.4)	0.826
Alcohol history*		5 (29.4)	15 (29.4)	0.984
Number of consultations				
- Mean number of visits (95%CI, SD)		3.9 (2.3–5.5, 3.2)	3.3 (2.7–3.96, 2.2)	0.257 [t(22*df*)= -0.664]
- Mean number of days between last visit and invitation to the study (95%CI, SD)		58.5 (-3.6–120.6, 120.8)	117 (56.5–177.6, 215.6)	0.085 [t(50*df*) = 1.391]

SD - Standard Deviation, CI – Confidence Interval, df – degrees of freedom, *data for only those who exhibited these symptoms is presented

### Questionnaire

Twelve (20%) out of the 60 participating clinicians returned the questionnaire. The results below relate to their responses. The low response rate is disappointing. As the responses were anonymised, we cannot comment on the characteristics of those who responded versus those who did not, nor can we know the reasons for failure to respond.

### A) identification of eligible cases

Ten (83%) clinicians confirmed they recalled being asked to refer, but only 4 (33%) had identified eligible cases. The reasons given for not identifying cases included: high workload, forgetting to refer, unfamiliarity with the eligibility criteria, time constraints, and also the fact that the eligible cases sought by the study were uncommon. Some specific responses are given below.

*“My clinical workload has recently been very very limited – I think I may have referred one person about 18 months ago – at least I recall discussing someone with your study team but perhaps they were not actually referred”. Clinician 12*.

*“I don’t recall having discussed this study & must apologise for this hence I have not been looking for eligible patients. Sorry”. Clinician 11*.

*“Lack of familiarity with eligibility; time constraints; geography (for patients)”. Clinician 10*.

*“I don’t tend to see these patients.” Clinician 1*.

### B) introducing the study

Among the 4 (33%) clinicians who had referred a case, all found the process of introducing the study clear.

*I did not find any difficulties. [The study nurse] is often present in the […] clinic so I could refer a patient in person. I have also emailed her about patients when she was not present. Both are very easy. Clinician 4*.

However, 2 (17%) highlighted some challenges they had faced such as building initial rapport (in time limited clinical consultations), the patient’s mental capacity to consent, and the lack of familiarity with the eligibility criteria.

*“Of the few patients that I have referred till date, I have not found it unduly difficult to introduce the study to them or to their NOK. Generally, I have felt more comfortable undertaking this - after having gained / achieved a degree of initial rapport with the family. Another challenge has been in relation to the patient’s mental capacity to consent towards involvement in the study”. Clinician 9*.

One clinician felt that introduction of the study or discussions of the study by the immediate clinical team would be preferred by the families of eligible patients.

*“…I suspect that some families might still prefer this initial introduction / scene setting discussion from a member of the current team (whether inpatient or outpatient basis); and before further direct contact is made from a study / project group member who they might not have met orb encountered”. Clinician 9*.

### C) referral

All the 12 clinicians who took part in the review indicated willingness to support the study, although to varying degrees with 8 (67%) of them mentioning they were clear on what was expected of them. For those unclear, various reasons for the lack of clarity were given, which included: education of staff, remembering the definition of atypical features, time constraints and need for further information.

*“Education of staff”. “Dependent on time constraints”. Clinician 10*.

*“To a degree; but one can sometimes find it difficult to remember some of the relevant or sought after ‘atypical’ features in real time”. Clinician 9*.

There were several suggestions made that could help improve referral of eligible patients to the study. These included regular email reminders, feedback to referrers, a referral template with anonymised examples, improve awareness of the study for clinicians, visible presence of the study team at the various facilities, and integration of the study with other research-oriented services.

*“….with other studies a regular e-mail reminder maybe once a month has been helpful in keeping things to the front of your mind”. Clinician 11*.

*“Ongoing periodic email communication – to serve as ‘gentle reminders’. Is there any scope to give potential referrers an idea of what a suitable referral looks like, e.g. using anonymised exemplars of cases that you have accepted ……response rates might (possibly) improve if potential referrers have an idea of a few anonymised ‘real’ or ‘accepted’ referrals that you have received.” Clinician 9*.

*“I simply don’t see enough patients and I think there needs to be an upstream register as relying on forgetful and distracted clinicians is usually unrewarding”. Clinician 11*.

*“Improved awareness for all clinicians, visible presence in [Hospital X].” Clinician 10*.

*“I suspect at the design stage more could have been done to integrate and introduce the work to MATS. In saying, that I don’t think MATS was the most ‘research orientated’ clinic in the universe but this is gradually changing….”. Clinician 12*.

### Actions taken

In an effort to improve recruitment, actions suggested by the review results were implemented. Awareness of the “case definition” of eligible cases was improved through the development of leaflets. Also, the study team presented in multi-disciplinary seminars (which also informed new clinicians), and encouraged them to get in touch if unsure of the case definition or the inclusion criteria. Workload challenges and forgetting to refer cases are closely interlinked, and have been shown to affect recruitment in research studies [12–14], therefore, these were addressed as follows in this study. Firstly, the amount of information about the study that the clinician needed to share with eligible patients was reduced and only needing a brief introduction and sharing of the study information sheet. Secondly, the study information sheet was given to participating clinics for distribution to eligible patients, and posters, for display in the relevant clinics. Thirdly, all clinicians were reassured during the site initiation visits that their involvement would be minimal. Fourthly, a monthly email reminder was instituted to check with participating clinicians whether they had seen any eligible patients. Some clinicians had suggested that the study nurse or study registrar should screen their local patient notes to determine patient eligibility, and also, draft a standard patient letter of invitation to the study that would be signed by the referring clinician. However, following consultation with the ethics board, the study team was advised that, that approach would be seen as a breach of confidentiality, therefore, it was not pursued further. Finally, arrangements were made with the local clinical teams to improve the visibility of the study team at the local sites. The study nurse attended ward rounds and memory clinics at the psychiatry of old age specialty, and was available for patient follow-up at the ARC. As evidence suggests, having a research nurse available to help clinicians recruit patients is likely to improve recruitment rates in research studies [15].

Despite significant efforts to increase recruitment, this was without success. In fact, after the review in the period between January 2018 and June 2019 (1 year and 6 months), there were only a further 9 referrals to the study (ARC: 1, all other services: 8).

## Discussion

As the study progressed, recruitment was lower than expected and there was uncertainty as to whether this simply reflected an initial over-estimation of cases fitting the eligibility criteria, or reflected methodological difficulties, or both. A review was undertaken to investigate the challenges that were involved in recruiting patients. While the study relied on the ability of the local clinicians to refer eligible cases, they may not have been able to do so for various reasons. Feedback from the clinicians highlighted some of these reasons, which included: unfamiliarity with/difficulty remembering the eligibility criteria, forgetting to refer, clinical workload, and time-limited consultations to allow for building rapport with the patients. Having identified some methodological difficulties, we took actions, where feasible, to try to improve referral and recruitment. Some of the problematic factors (such as a clinician’s high workload) were beyond our influence and there is likely to be some reluctance from some patients or families to engage with research in the context of the elderly with cognitive problems. The relatively low response rate is disappointing and limits our understanding of possible barriers to recruitment. The data from those who responded cited clinical workload, forgetting and time-limitations, all of which might be factors in failure to respond to the questionnaire. It is possible that limited interest in the aim of the study also contributed.

Overall, the actions taken did not lead to an improvement; in the period following this review numbers were low. In assessing this, there are two factors to be taken into account. Firstly, the original study nurse unexpectedly left in March 2018 and it took 4 months to recruit and appoint a second study nurse who took up the post in August 2018; during this period other staff maintained the study but it was not possible to maintain the same degree of contact with the participating clinical service centres, which could have had a negative impact on the study. Secondly, the initial referral rate was expected to be higher as there were likely to be existing patients already known to the clinical services, with later referrals being only newly presenting patients. It also remains uncertain as to whether our initial estimates of eligible cases were excessive. However, recruitment remained low and 75% of the referred, eligible patients declined to take part. Of those referred patients, there were no definite major differences in clinical characteristics between those recruited and those not recruited, with low numbers making firm comments difficult. While it might be expected that older patients and ones without a spouse might be more difficult to recruit, just over half of the non-recruited were in the younger end of our age group (64–69) and nearly 59% had a spouse as main carer. It is notable that most referrals (72 cases) came from one centre-the ARC, with a total of 47 from all the other participating centres. This has two explanations. Firstly, one might expect better recruitment from a specialised clinic with a dedicated research function, on the same site as the study office, than from other, busy, more peripheral units, with an essentially clinical function. Secondly, atypical cases are more likely to be referred to a specialised clinic like the ARC.

This study probably represents the best that can be done in the absence of a major study, such as a comprehensive review of all elderly brain illness with a very high autopsy rate. This would be a significant undertaking and unfortunately impractical in terms of available resources. The study represented a focussed approach in one health care region. It did not identify any previously unsuspected cases of CJD (sporadic or variant) but, even within its restricted scope, there were methodological problems which mean that cases could possibly have been missed.

While the question of case under-ascertainment remains important, one practical suggestion to improve recruitment could involve searching digital records (or anonymised derivatives) held in NHS Lothian to identify eligible patients, who would then either go through the clinician route for further assessment or the data used in a desk based review. An interesting possibility is the development of relatively non-invasive diagnostic tests for prion disease, based on the highly specific detection of abnormal prion protein, which could be used for screening appropriate patients. Potential tests include blood, urine, skin biopsy and nasal brushing tests [16–19].

The study, overall, with the pathological data (described elsewhere), could be taken as a partial reassurance that large numbers of sporadic or variant CJD cases are not being missed in the over 65 years population. Pathological ascertainment remains the most attractive alternative approach but, given the very low autopsy rates in elderly dementia, this is also problematic. If the fall in annual mortality rate of sCJD in the very elderly is not simply due to case under-ascertainment, then perhaps other research should be designed to explore other explanations. Similarly, the relatively small number of vCJD cases and blood-transmitted infections may also not be due to case under-ascertainment and research into other explanations could be considered.

## Conclusion

It was recognised that enhanced CJD surveillance in the elderly might pose significant methodological problems and this study was designed mainly as a feasibility study. Despite the study representing the best idea that was financially, ethically and practically possible, with an early review and subsequently improved protocol, few cases were recruited. The unplanned absence of a study nurse for a 4-month period could have impacted recruitment, but not to a large degree. Searching digital records (or anonymised derivatives) to identify eligible patients could be explored, who would then either go through the clinician route for further assessment or the data used in a desk based review.

## Data Availability

The data is held by the NCJDRSU as part of their surveillance projects. The NCJDRSU is an internationally recognised World Health Organisation reference centre and European Centre for Disease Control hub for diagnosis of all forms of human prion disease and has substantial expertise in prion disease surveillance and clinical and laboratory research in neurology, neuropathology, brain imaging and biochemical investigations in relation to dementing illness (www.cjd.ed.ac.uk). Enquiries to access the data can be made to NCJDRSU (https://www.cjd.ed.ac.uk/contact-us), which will be considered on a case by case basis in line with the NCJDRSU Data Protection and Security Code of Practice.

## List of abbreviations

ARC: Anne Rowling Clinic
BSE: Bovine Spongiform Encephalopathy
CJD: Creutzfeldt - Jakob disease
CDC-DART: Cognitive Disorders Clinic Diagnosis, Audit, Research and Treatment Register
MOE: Medicine of the Elderly
NHS: National Health Service
POA: Psychiatry of Old Age
sCJD: Sporadic Creutzfeldt-Jakob Disease
UK: United Kingdom
vCJD: Variant Creutzfeldt-Jakob Disease
